# Supplementary material for: Analyzing protein dynamics from fluorescence intensity traces using unsupervised deep learning network
Source: Commun Biol. 2020 Nov 12;3:669. doi: 10.1038/s42003-020-01389-z (PMC7665068; doi:10.1038/s42003-020-01389-z)
Supplement: Supplementary file 1 — Supplementary Information [file 42003_2020_1389_MOESM1_ESM.docx]

**Supplementary Information**

**Analyzing protein dynamics from fluorescence intensity traces using unsupervised deep learning network**

Jinghe Yuan,^1*^ Rong Zhao,^2^ Jiachao Xu,^1^ Ming Cheng,^1^ Zidi Qin,^3^ Xiaolong Kou,^1^ Xiaohong Fang^1,3*^

1. Key Laboratory of Molecular Nanostructure and Nanotechnology, CAS Research/Education Center for Excellence in Molecular Sciences, Institute of Chemistry, Chinese Academy of Sciences, Beijing 100190, China

2. Division of Chemical Metrology and Analytical Science, National Institute of Metrology, Beijing 100029, China

3. University of Chinese Academy of Sciences, Beijing 100049, China

**Table of Contents:**

| **1. SUPPLEMENTARY METHODS** | **S2** |
| --- | --- |
| **2. SUPPLEMENTARY Tables** | **S3** |
| **3. SUPPLEMENTARY Figures** | **S4** |
|  |  |

1. **SUPPLEMENTARY METHODS**

**data synthesizing**

To train, validate and test the network, we synthetized fluorescence intensity traces datasets (72,000 traces with hypothetical maximum state 10 and minimum segment length 5 frames for photobleaching event counting, 60,000 traces with hypothetical maximum state 5 and minimum segment length 5 frames for dynamic finding). We synthesizing the datasets by referring the experimental fluorescence intensity traces. For example, we analyzed the distribution of the experimental fluorescence intensity in one experiment with gaussian mixture model (figureS1(a)), and found the means and the variances of the background sequence and the fluorescence intensity sequence of single step. By setting background mean and variances as 25 ($\mu_{1}$) and 49（$\sigma_{1}^{2}$） respectively, and setting the mean and variances of fluorescence intensity of single step as 36（$\mu_{3}-\mu_{2}$） and 224（$\sigma_{2}^{2}$） respectively, we synthetized the fluorescence intensity traces. By assuming the photoblinking events belong to binomial distribution and setting the success number $N=3$ and success probability $p=0.98$, we considered the effects of photoblinking also. Figure S1(b) is an example of fluorescence photonbleaching trace.

1. **SUPPLEMENTARY TABLES**

**Supplementary Table 1.** The detailed parameters of the framework, where the values(C) is for photobleaching event counting and values(D) is for dynamic finding.

| **Parameters** | **values(C/D)** |
| --- | --- |
| Maximum hidden state number | 10/5 |
| Feature dimension of input | 1 |
| Hidden LSTM unit number of the discriminator | 32 |
| Hidden LSTM unit number of the generator | 16 |
| Maximum epochs | 60/34 |
| Minibatch size | 8 |
| Momentum factor | 0.9 |
| Initial Learning Rate | 0.001 |
| Learning rate dropping period | 2000 |
| Learning rate dropping factor | 0.5 |
| Weight gradient threshold | 1 |
| Decay factor | 0.0001 |
| Validating Period | 100 |

1. **SUPPLEMENTARY figures**

**Supplementary Figure 1**


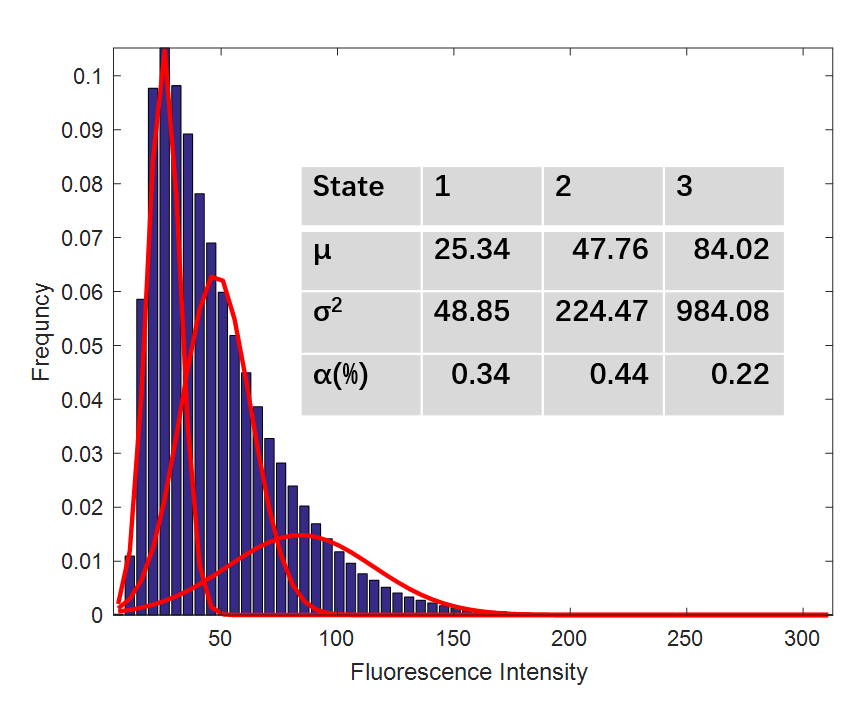


(b)

(a)

**Photoblinking at t_49_-t_52_**

**Supplementary Figure 1.** (a) The distribution of the experimental fluorescence intensity analyzed with gaussian mixture model. (b) A synthetized fluorescence photobleaching trace in consideration of the three types of noises (Poisson noise, Gaussian Noise and photo-blinking).

**Supplementary Figure 2(a)**

**
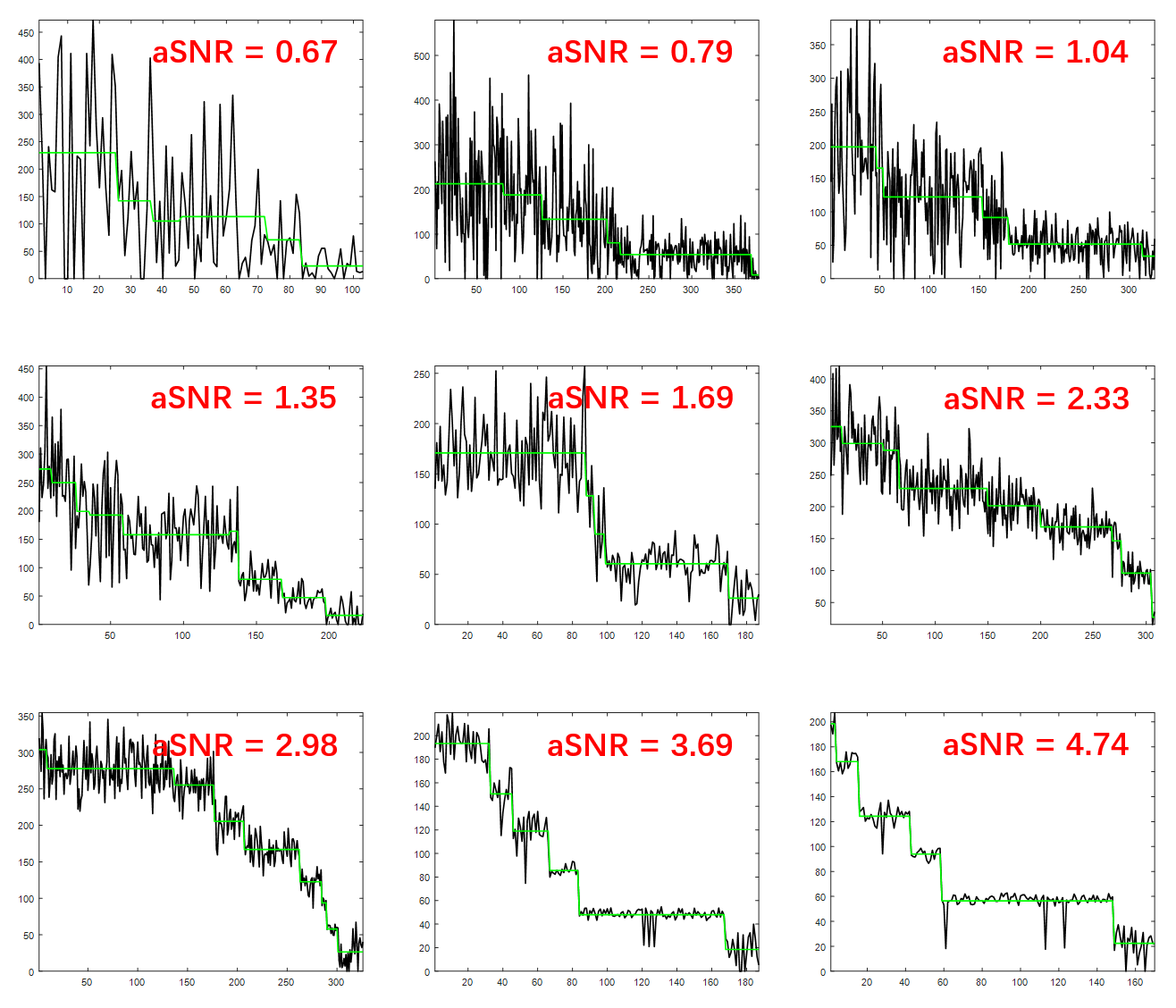
**

**Supplementary Figure 2(a).** Synthetized fluorescence intensity traces (black lines) with different signal to noise ratios (aSNR) for fluorescence photobleaching counting, where the green broken lines are the synthetized state paths.

**Supplementary Figure 2(b)**


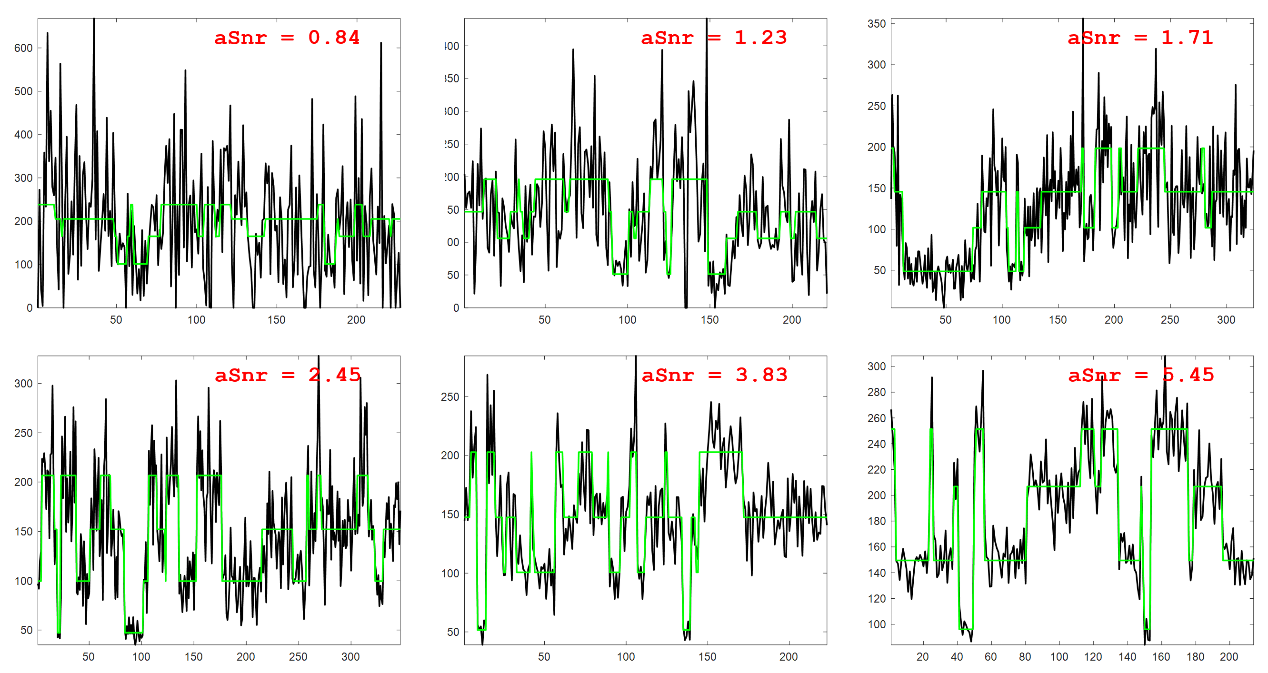


**Supplementary Figure 2(b).** Synthetized fluorescence intensity traces (black lines) with different signal to noise ratios (aSNR) for dynamic finding, where the green broken lines are the synthetized state paths.

**Supplementary Figure 3**

**Supplementary Figure 3.** The loss curves of training and validating the framework.

**Supplementary Figure 4**

**Supplementary Figure 4.** The normalized confusion matrixes of predicted accuracies under aSNR 3.69. The predicted accuracy of the framework defined as the lowest accuracy of the 10 states, namely, 0.806.

**Supplementary Figure 5**

**Supplementary Figure 5.** The classification accuracies of 50 hidden states under 7 different signal-noise ratios (aSNR) with the framework.
