# Supplementary material for: Analyzing protein dynamics from fluorescence intensity traces using unsupervised deep learning network
Source: Commun Biol. 2020 Nov 12;3:669. doi: 10.1038/s42003-020-01389-z (PMC7665068; doi:10.1038/s42003-020-01389-z)
Supplement: Supplementary file 3 — Reporting Summary [file 42003_2020_1389_MOESM3_ESM.pdf]

nature research

Corresponding author(s): Jinghe Yuan
Last updated by author(s): Sep 21, 2020

Reporting Summary

Nature Research wishes to improve the reproducibility of the work that we publish. This form provides structure for consistency and transparency in reporting. For further information on Nature Research policies, see our Editorial Policies and the Editorial Policy Checklist.

Statistics

For all statistical analyses, confirm that the following items are present in the figure legend, table legend, main text, or Methods section.

n/a	Confirmed	
☐	☒	The exact sample size (n) for each experimental group/condition, given as a discrete number and unit of measurement
☐	☒	A statement on whether measurements were taken from distinct samples or whether the same sample was measured repeatedly
☒	☐	The statistical test(s) used AND whether they are one- or two-sided<br>*Only common tests should be described solely by name; describe more complex techniques in the Methods section.*
☒	☐	A description of all covariates tested
☒	☐	A description of any assumptions or corrections, such as tests of normality and adjustment for multiple comparisons
☐	☒	A full description of the statistical parameters including central tendency (e.g. means) or other basic estimates (e.g. regression coefficient) AND variation (e.g. standard deviation) or associated estimates of uncertainty (e.g. confidence intervals)
☒	☐	For null hypothesis testing, the test statistic (e.g. F, t, r) with confidence intervals, effect sizes, degrees of freedom and P value noted<br>*Give P values as exact values whenever suitable.*
☒	☐	For Bayesian analysis, information on the choice of priors and Markov chain Monte Carlo settings
☒	☐	For hierarchical and complex designs, identification of the appropriate level for tests and full reporting of outcomes
☒	☐	Estimates of effect sizes (e.g. Cohen's d, Pearson's r), indicating how they were calculated

Our web collection on statistics for biologists contains articles on many of the points above.

Software and code

Policy information about availability of computer code

Data collection	Andor iQ (1.10.3) was used for EMCCD controlling during TIRFM imaging.
Data analysis	Custom Matlab codes in the current study have been submitted with this submitting.

For manuscripts utilizing custom algorithms or software that are central to the research but not yet described in published literature, software must be made available to editors and reviewers. We strongly encourage code deposition in a community repository (e.g. GitHub). See the Nature Research guidelines for submitting code & software for further information.

Data

Policy information about availability of data

All manuscripts must include a data availability statement. This statement should provide the following information, where applicable:
- Accession codes, unique identifiers, or web links for publicly available datasets
- A list of figures that have associated raw data
- A description of any restrictions on data availability

All the data of this manuscript were achieved in Dryad by the link: https://datadryad.org/stash/share/9liw8QzlGcUgwdIz5nahbp6wVjXhZvD03ZKp_w5bURg.

Field-specific reporting

Life sciences study design

All studies must disclose on these points even when the disclosure is negative.

Sample size	For photobleaching event counting, 72000 traces were synthesized for net training and 7200 for validating and testing respectively. For dynamic finding, 60000 traces were synthesized for net training and 6000 for validating and testing respectively. The determination of the synthetic sample size is according to the convergence of the nets.<br>For photobleaching event counting from the experimental data, 2688 traces from 24 resting MCF7 cells and 2553 traces from 21 MCF7 cells with TGF-β stimulation were used. For dynamic finding from the experimental data, 1323 traces without TGF-β stimulation, 5694 traces with TGF-β stimulation but without Nystatin treatment, and 4459 traces with TGF-β stimulation and Nystatin treatment. The experimental sample size was determined by observing that more sample did not alter the results obviously.
Data exclusions	No data were excluded.
Replication	Bootstrap analysis was taken to measure the repeatability, the mean and the standard deviation were demonstrated in the corresponding figures.
Randomization	Bootstrap analysis was taken to measure the repeatability, in which 72% of the whole samples were selected randomly in once test.
Blinding	The investigators were blinded during data collection.

Reporting for specific materials, systems and methods

We require information from authors about some types of materials, experimental systems and methods used in many studies. Here, indicate whether each material, system or method listed is relevant to your study. If you are not sure if a list item applies to your research, read the appropriate section before selecting a response.

Materials & experimental systems

n/a	Involved in the study
☒	☐ Antibodies
☐	☒ Eukaryotic cell lines
☒	☐ Palaeontology and archaeology
☒	☐ Animals and other organisms
☒	☐ Human research participants
☒	☐ Clinical data
☒	☐ Dual use research of concern

Methods

n/a	Involved in the study
☒	☐ ChIP-seq
☒	☐ Flow cytometry
☒	☐ MRI-based neuroimaging

Eukaryotic cell lines

Policy information about cell lines

Cell line source(s)	HeLa and MCF7 cells were purchased from Cell Resource Center, IBMS, CAMS/PUMC (Beijing, China)
Authentication	The STR analysis for human cell line authentication showed that there was no tri-allelic phenomenon at each locus, and no cross-contamination of human cells was found in the cells. The cell DNA typing found 100% matching HeLa and MCF7 cells in the ATCC, DSMZ and JCRB cell banks.
Mycoplasma contamination	Hela and MCF7 cells was tested negative for mycoplasma contamination
Commonly misidentified lines (See ICLAC register)	No commonly misidentified cell lines used.
